# A Retrospective Study of the Ultrasound Imaging Characteristics of Juvenile Xanthogranuloma

**DOI:** 10.3390/jcm15062134

**Published:** 2026-03-11

**Authors:** Hong Wang, Xiaoyan Peng, Yujia Yang

**Affiliations:** Department of Medical Ultrasound, West China Hospital of Sichuan University, 37 Guoxuexiang, Chengdu 610041, China; wanghong@wchscu.cn (H.W.);

**Keywords:** juvenile xanthogranuloma, ultrasound, hypoechoic, multimodal imaging

## Abstract

**Objectives:** To strengthen the recognition of juvenile xanthogranuloma (JXG) by analyzing ultrasound findings. **Methods:** This study retrospectively enrolled these patients with pathologically confirmed JXG from January 2011 to March 2025. The clinical, imaging, pathological features, and prognosis of all included patients were analyzed. All the imaging features were evaluated in consensus by two radiologists. **Results:** Fourteen patients were included in the study. A total of 78.6% presented with solitary masses. The age of the patients ranged from 2 months to 48 years. Those aged ≤1 year accounted for 64.3% of the sample. The lesions were predominantly located on the head and face, and the skin of most patients was yellowish-orange. The ultrasound manifestations are mostly hypoechoic masses with clear boundaries and regular shapes. Contrast-enhanced ultrasound shows a slight homogeneous enhancement, and on shear wave elastography, it appears to be relatively hard. **Conclusions:** JXGs are more common in infants or young children and present with yellowish-orange, cutaneous lesions. Ultrasound revealed homogeneous, well-circumscribed, regular hypoechoic nodules. Multimodal imaging may be helpful for preoperative diagnosis.

## 1. Introduction

Juvenile xanthogranuloma (JXG) is a rare benign histiocytic proliferative disorder that is classified as non-Langerhans cell histiocytosis (N-LCH) [1]. JXG mainly originates from dermal dendrocytes. Its typical pathological feature is localized infiltration of lipid-laden macrophages, often accompanied by Touton giant cells. Although the pathogenesis has not been fully elucidated, studies have shown that it may be closely related to genetic factors (especially mutations in the MAPK pathway), immune dysfunction, and abnormal inflammatory responses [2]. Epidemiological data show that the overall incidence of JXG is extremely low. A large-sample cohort study from the Kiel Pediatric Tumor Registry reported that its incidence was only 0.52% [3].

This disease mainly occurs in infancy. Approximately 85% of the patients develop lesions within the first year after birth, whereas 10% of cases occur in adulthood, making adult-onset JXG relatively rare. The male incidence rate is slightly higher than that of females, with a sex ratio of approximately 1.2:1 to 1.5:1. It usually progresses in a self-limiting manner [4,5]. owing to its low incidence rate and lack of specific clinical manifestations, clinical physicians have an inconsistent understanding of its diagnosis and differential diagnosis. In particular, atypical cases are easily confused with other cutaneous and subcutaneous tissue lesions, which pose great challenges for clinical diagnosis and treatment.

On the basis of the scope and location of the lesion, JXG can be clearly classified into two clinical subtypes, and there are significant differences in their clinical characteristics, prognoses, and management strategies: (1) Cutaneous juvenile xanthogranuloma (CJXG), accounting for approximately 80% of all cases. It is characterized by nodular cutaneous lesions limited to the superficial layer of the dermis and subcutaneous tissue, and is predominantly distributed on the head, neck, and trunk, without the involvement of other organs. CJXG does not involve other internal organs, and the course of the disease is self-limiting. Most lesions will disappear on their own within 3 to 6 years after the onset of the disease. Generally, no special treatment is needed; only regular follow-up observation is sufficient [6]. (2) Systemic juvenile xanthogranuloma (SJXG), which is uncommon and can occur with or without CJXG. The eye is the most prevalent site of SJXG involvement, while the involvement of other anatomical sites, such as the central nervous system, lungs, liver, spleen, kidneys, and bone, has also been reported in clinical and research studies [7,8]. Although JXG usually has a self-limiting course, SJXG may cause serious complications (such as glaucoma, hepatosplenomegaly, or intracranial mass effects) due to multiorgan involvement [9,10], and the clinical management of JXG urgently requires multidisciplinary cooperation and comprehensive, accurate assessment.

Currently, most studies on JXG are case reports. In existing research, descriptions of imaging manifestations of cutaneous and extracutaneous multiorgan involvement are scarce, and imaging studies have focused mainly on computed tomography or magnetic resonance imaging [11,12]. Ultrasound examination, as a noninvasive, convenient, and repeatable imaging diagnostic method, has the advantages of simple operation, no ionizing radiation, and low cost. It plays an irreplaceable role in the diagnosis, differential diagnosis, and follow-up of cutaneous and subcutaneous tissue lesions. It can clearly display the location, size, shape, boundary, internal echogenicity, and blood flow signals of the lesion, providing important imaging clues for clinical diagnosis. However, at present, there is no consensus on the ultrasound imaging features of JXG in clinical practice. Ultrasound physicians have an insufficient understanding of its imaging manifestations, making it difficult to accurately identify and classify JXG through ultrasound examinations, and thus are unable to provide reliable imaging support for clinical diagnosis and treatment.

Therefore, this study retrospectively analyzed the ultrasonic imaging characteristics of patients with JXG confirmed by surgical pathology at our center. We recorded and analyzed the ultrasound indicators such as the location, size, shape, boundary, internal echogenicity, and blood flow signals of the lesion. Combined with the patients’ clinical data and pathological results, we explored the application value of ultrasound examination in the diagnosis, classification, and differential diagnosis of JXG. The aim of this study was to enhance the understanding and diagnostic level of this disease and provide a more comprehensive imaging basis for clinical diagnosis, treatment planning, and prognosis assessment.

## 2. Materials and Methods

### 2.1. Patient Data Acquisition

Ethical approval for the present retrospective study was granted by the Institutional Review Board of our affiliated institution. Fourteen patients with pathologically verified JXG were enrolled in this study, and their clinical data were collected retrospectively and consecutively between January 2011 and March 2025. Before any imaging examinations were performed, informed written consent was acquired from all enrolled patients (or their legal guardians for pediatric patients), and a detailed explanation of examination-related potential risks and procedural complications was provided. Comprehensive clinicoradiological characteristics and treatment details for all patients were retrieved from the electronic medical records.

### 2.2. Image Acquisition

For patients with multiple lesions, only the largest lesion was analyzed and reported in the ultrasound examination. Conventional ultrasound scans (B-mode, color Doppler flow imaging (CDFI), and power Doppler imaging (PDI)), contrast-enhanced ultrasound (CEUS), and shear wave elastography (SWE) images were available for review. Ultrasound examinations were carried out with an iU22 ultrasound system equipped with an L12-5 (5–12 MHz) linear array transducer (Philips Medical Systems, Royal Philips, the Netherlands), Siemens Acuson Oxana2 US equipment (Siemens Medical Solutions, Mountain View, CA, USA) with L18-6 (6–18 MHz) and L9-4 (4–9 MHz) linear array transducers, and a Resona7 ultrasonic diagnostic system (Mindray Medical International Co., Ltd., Shenzhen, China) configured with an L14-6 linear array transducer (frequency: 6–14 MHz). All the examinations were performed by radiologists with >5 years of experience in superficial tissue ultrasonography. Relevant ultrasound parameters (gain, depth, focal position) were optimized on the basis of the radiologist’s clinical experience to ensure high-quality visualization of the target lesions. Conventional ultrasound was completed for all included patients, and systematic assessment of ultrasonic findings was carried out, including lesion location, boundary definition (clear, ill-defined), morphological regularity (regular, irregular), size (maximum longitudinal diameter of the lesion’s largest cross-section), echo characteristics (hypoechoic, hyperechoic, or isoechoic), internal echogenicity (homogeneous, heterogeneous), calcification and peripheral acoustic halo presence, CDFI and PDI signals, and the spatial correlation between the lesion and surrounding anatomical structures.

Among the included patients, one patient underwent SWE and CEUS in addition to conventional ultrasound examination as described above, and the dynamic imaging videos were analyzed. During SWE, the transducer was held gently without applying pressure over the target lesion. The patient was instructed to perform breath-holding to avoid motion artifacts. The radiologist then switched to SWE mode and maintained complete transducer stability for 3–5 s until the color-coded overlay was fully displayed. The shear wave velocity on SWE was quantified within the designated region of interest of the lesion. This measurement was repeated three times for the lesion, and the average value of shear wave velocity was recorded. The elasticity hardness of the lesion is semi-quantitatively and quantitatively displayed using a color scale for elastic modulus. This scale maps the hardness from blue (soft) to red (hard) on a continuous color spectrum, and is supplemented by shear wave velocity (m/s) as a quantitative reference. After SWE was completed, the patient underwent CEUS. On CEUS, CEUS along with a dual-screen mode (allowing simultaneous display of grayscale ultrasound and CEUS images on the monitor) was utilized for real-time contrast-specific imaging at a low mechanical index (the mechanical index setting was 0.09 in Siemens Acuson Oxana2 US equipment). A dose of 2.4 mL of SonoVue (Bracco, Milan, Italy) suspension was injected intravenously through the patient’s cubital vein, followed by a 5 mL saline flush. The timer was activated immediately after the completion of contrast agent administration. The target lesion was observed continuously, and dynamic images were stored in real time for 180 s. The dynamic contrast enhancement patterns of the lesion were evaluated and analyzed.

### 2.3. Statistical Analysis

All the statistical analyses were performed using IBM SPSS Statistics Version 27 (IBM Corporation, Armonk, NY, USA). This study employed descriptive statistical methods to summarize the basic characteristics of the study samples, mainly using frequency and percentage to represent the distribution of categorical variables.

## 3. Results

### 3.1. Clinical Manifestations of Patients

**Figure 1:**
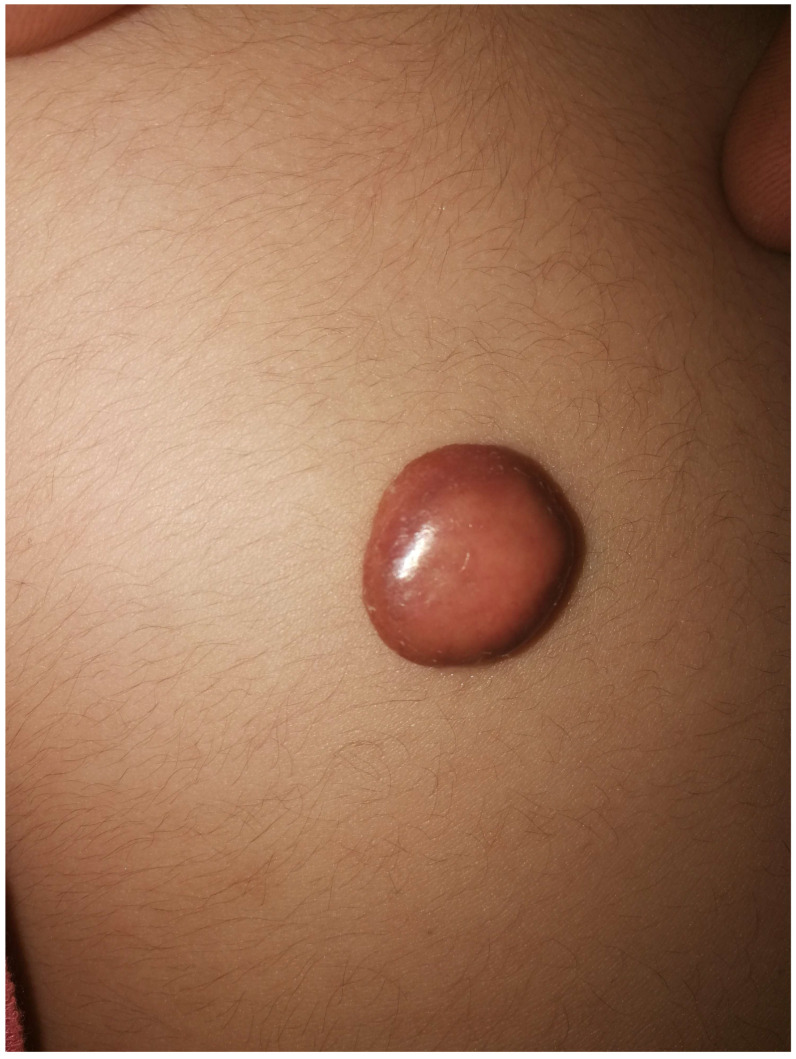
Physical examination revealed a solitary, well-circumscribed, deep-red subcutaneous nodule measuring approximately 15 × 15 mm in the left back region. The lesion protruded above the skin surface, and it was not tender on palpation.

A total of fourteen patients were included in the study. The male-to-female ratio was 1:1. The age of the patients ranged from 2 months to 48 years, with nine patients (64.3%) being ≤1 year old. Three patients presented with multiple lesions (21.4%), whereas the remaining patients (11/14, 78.6%) presented with solitary masses. The lesions were predominantly located on the head and face (eight patients), followed by the back (two cases), shoulders (two patients), and left breast, right thigh, and left neck (one patient each). Macroscopically, the overlying skin of the lesions showed yellowish-orange discoloration in 10 cases, deep red in three cases (Figure 1), and brownish in one case. Only one lesion was identified incidentally during ultrasound examination (Case 13), whereas all remaining patients presented with clinically palpable masses, and no associated clinical symptoms were observed in any patient. The results of blood examinations, including complete blood count, renal function tests, and liver function tests, were all within the normal reference range.

### 3.2. Ultrasonographic Features

**Figure 2:**
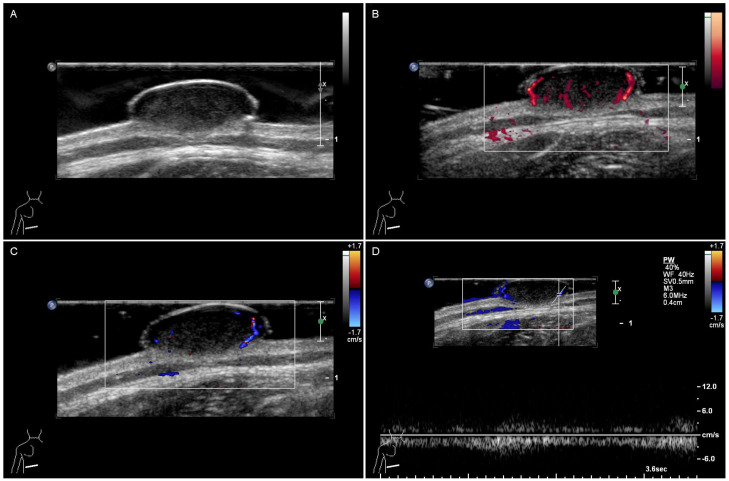
Conventional ultrasonographic features of a typical JXG lesion: (**A**) B-mode imaging reveals a hypoechoic mass with well-defined borders and a regular shape; (**B**,**C**) power Doppler imaging and color Doppler flow imaging detect internal and peripheral vascularity with scattered flow signals; (**D**) pulsed-wave Doppler analysis confirms a venous flow pattern characterized by continuous, low-velocity waveforms.

All 14 patients underwent conventional ultrasound. Case 13 was suspected of having a malignant breast tumor on the initial conventional ultrasound, and thus underwent CEUS and SWE examinations as a clinical recommendation. All cases were located in the skin or in the skin and the superficial subcutaneous layer. The maximum diameter of the lesions ranged from 6 mm to 38 mm. All lesions were round or oval in shape, with 12 cases (85.7%) having clear boundaries and regular shapes, and two cases (14.3%) having ill-defined and irregular shapes. The lesions of the fourteen patients were all hypoechoic lesions. Homogeneous internal echoes were observed in 11 lesions (78.6%), whereas heterogeneous internal echoes were noted in only three cases (21.4%). Only Case 13 showed calcification under ultrasound. On CDFI, eight lesions (57.1%) presented internal blood flow signals, whereas five lesions (35.7%) presented no obvious internal blood flow signals. No obvious peripheral blood flow signals were observed in the lesions. The PDI results revealed that five lesions (35.7%) presented both internal and peripheral flow signals, six lesions (42.9%) presented solely internal vascularization, and three lesions (21.4%) presented an absence of discernible flow signals both within and surrounding the lesion. The conventional ultrasound manifestations of all included patients are shown in Table 1. A typical ultrasonic image is shown in Figure 2.

**Figure 3:**
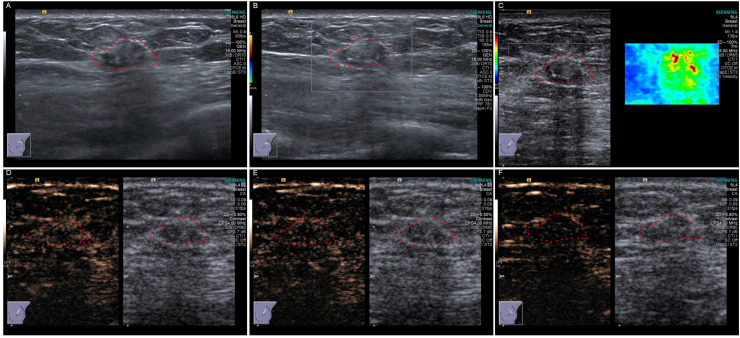
Multimodal ultrasonographic manifestations of Case 13 (breast JXG): (**A**) B-mode ultrasound showed an irregular hypoechoic mass with ill-defined margins and internal calcification. (**B**) Color Doppler flow imaging revealed no significant internal and peripheral vascularity. (**C**) Shear wave elastography depicted tissue stiffness characteristics: based on the color scale of elastic modulus, the image showed that the interior of the lesion was predominantly orange and red signals, which form a clear contrast with the surrounding normal glandular tissue (which appears blue or green, indicating a soft texture). Quantitative analysis results indicated that the average shear wave velocity of the lesion was 6.84 m/s, much higher than that of the adjacent glandular tissue. This hardness manifestation conforms to the imaging features of a solid, relatively hard lesion in the BI-RADS classification, suggesting that the lesion has a relatively high tissue hardness and a higher possibility of malignancy. (**D**–**F**) Contrast-enhanced ultrasound sequences showing dynamic perfusion patterns in the arterial phase (25 s) (**D**), venous phase (40 s) (**E**), and delayed phase (120 s) (**F**). CEUS revealed slight homogeneous enhancement. The area delineated by the red dashed circle represents the tumor region.

In Case 13, the initial conventional ultrasound examination raised suspicion of a malignant breast tumor. Consequently, on the basis of the clinical recommendation, this patient subsequently underwent CEUS and SWE in addition to the standard ultrasound evaluation. The conventional ultrasound findings in this case included a hypoechoic nodule with an ill-defined margin and an irregular shape, heterogeneous internal echogenicity, and no appreciable internal and peripheral blood flow on CDFI. These features were consistent with a suspicion of malignancy. Further SWE and CEUS were subsequently conducted, and were completed successfully in this patient with satisfactory imaging quality. CEUS revealed an ill-defined margin and irregular shape of the lesion. Slight enhancement was observed without significant post-enhancement enlargement. The diagnosis was classified as BI-RADS category 3 (possible benign lesion, malignant risk less than 2%). However, the SWE results revealed a shear wave velocity of 6.84 m/s, which is indicative of suspected malignancy (Figure 3).

### 3.3. Histopathological Features and Follow-Up

Fourteen patients underwent surgical resection of the lesions. Pathological assessment revealed no neoplastic involvement at the incisal margin of the resected tissue specimens submitted by any patient. Microscopically, the lesion was characterized by the proliferation of histiocytes, foamy macrophages, and multinucleated giant cells (including classic Touton giant cells), accompanied by cytoplasmic vacuolation. The background stroma contained scattered small lymphocytes, plasma cells, and occasional individual eosinophils. Immunohistochemical staining revealed positive expression of CD68/PGM1 and CD163. CD1a and S-100 were negative. All fourteen cases were pathologically diagnosed with CJXG.

## 4. Discussion

JXG, which is a rare benign lesion with a self-limiting tendency, was first reported by Adamson in 1905, and its pathogenesis remains unclear [13]. Current evidence [14] suggests that it may be associated with nonspecific tissue damage triggered by viral infections, vaccinations, genetic factors, or other etiologies, leading to a proliferative response of dendritic cells and macrophages. CJXG is the most common type of this disease, and predominantly affects the scalp, face, and trunk. Most patients can regress spontaneously or tend to stabilize within 3–6 years. The disease has a long course, which is likely to cause anxiety among parents of pediatric patients. The main treatment method is surgical resection [15,16]. Currently, high-frequency ultrasonography is widely used in the evaluation of superficial masses and also has important value in the diagnosis and follow-up of JXG. This study summarized the clinical manifestations, ultrasound features, and pathological findings of 14 cases of CJXG, aiming to provide a reliable basis for the diagnosis of this disease by high-frequency ultrasound.

In this study, all reported cases involved CJXG, which was localized to the cutaneous and subcutaneous layer. All lesions presented as hypoechoic masses, which is consistent with previous reports [17,18]. Among the 14 patients studied, 11 lesions (78.6%) exhibited regular shape, well-defined margin, and homogeneous internal echogenicity. The remaining three lesions demonstrated heterogeneous internal echogenicity. Histopathological examination of these three lesions revealed patchy proliferation of mononuclear cells/histiocytes, foamy macrophages, and multinucleated giant cells, including Touton-type giant cells. This finding suggested that the observed sonographic heterogeneity is likely attributable to the admixture of these distinct cellular components. Our research revealed that the manifestations of blood flow signals within the lesion are relatively diverse, indicating that the application of CDFI and PDI in the diagnosis of JXG needs to be further explored and validated in larger sample studies.

In addition to ultrasonography, the dermoscopic manifestations of JXG have also been reported in clinical practice [19,20]. Its dermoscopic features include the “setting-sun” sign, pale yellow globules, subtle pigment networks, whitish streaks, and branched linear or dotted vessels. These characteristic manifestations are considered to be associated with the maturation stage of the lesions [21]. The sunset sign can be observed in both the early developmental and fully mature stages of the lesions. In fully mature lesions, peripheral erythema fades while yellow granular structures become more prominent. In late degenerative lesions, distinct whitish streaks are detectable. This highlights key differences in the detection focus of the two modalities. Ultrasonography primarily reflects the deep structural characteristics and blood flow of lesions. Its advantage lies in the ability to assess deep dermal and subcutaneous lesions, helping to identify deep infiltration [22]. Dermoscopy focuses on surface pigment distribution patterns and vascular morphology [19]. Its advantage is that it can quickly conduct clinical screening through unique features such as the “setting-sun” appearance, indicating the maturity of the lesion. Despite these differences, both techniques can accurately characterize JXG disease progression from distinct dimensions. The combined application of these two modalities is expected to have important clinical value in the early identification, clinical classification, and differential diagnosis of JXG.

According to the literature [23], adult-onset CJXG represents approximately 15% of all diagnosed JXG cases, typically presenting with a single lesion in the head or neck region. In contrast to the juvenile form, spontaneous regression rarely occurs in adult patients, and approximately 5% of adult CJXG cases are identified in individuals aged 60 to 70 years. Herein, we report for the first time the conventional ultrasound, CEUS, and SWE findings of a “juvenile-type” xanthogranuloma located in the subcutaneous tissue of the breast in a 48-year-old female patient (Case 13). In this case, conventional ultrasound revealed a hypoechoic nodule with an ill-defined margin and irregular shape, accompanied by calcification. CDFI revealed no appreciable flow signals. CEUS revealed slight homogeneous enhancement, suggesting a possible benign lesion. SWE revealed an elevated shear-wave velocity (velocity = 6.84 m/s), suggesting increased stiffness and raising suspicion of malignancy. Histopathological examination revealed the presence of sheet-like arrangements and local aggregations of medium to large-sized round tissue cells within the superficial subcutaneous tissue. The focal areas exhibited epithelioid cell morphology. The background contained scattered small lymphocytes, plasma cells, and occasional eosinophils. Small lymphoid aggregates, focal necrosis, and apoptotic bodies were also observed. Immunohistochemistry demonstrated positivity for CD68/PGM1, CD163 and CD4, with ALK-V, OCT2, S-100, CD1a, Langerin, CyclinD1 and CXCL13 being uniformly negative. p53 showed weak focal staining, and Ki-67 labeled approximately 3% of the lesional nuclei. In the KNBP mutation profiling using ADx-ARMS technology, no pathogenic variants were detected in the KRAS, NRAS, BRAF, or PIK3CA loci, with an analytical sensitivity threshold of a 1% mutant allele frequency. When morphological features, immunohistochemical staining results, and clinical history were integrated, the diagnosis was consistent with that of JXG, despite the patient’s advanced age, atypical lesion location, and suspicion of malignancy on both conventional and SWE ultrasound. This finding further suggests that CEUS may offer superior diagnostic value in the evaluation of JXG, especially for atypical cases.

Although this study covered a time span of over 14 years, it is undeniable that the technical limitations in the early stages had a significant effect on the completeness of this study cohort. Specifically, retrospective analysis showed that CEUS and SWE were not widely applied in clinical practice in the early stage of the study because of the limitations of hardware conditions and the clinical workflow at that time. Therefore, except for one patient who completed a full multimodal ultrasound examination (Case 13), the remaining cases were mainly evaluated by conventional B-ultrasound and Doppler imaging. However, this single case with complete multimodal ultrasound data provided valuable insights. The CEUS and SWE results of this patient revealed opposite indications for benign/malignant differentiation, highlighting the potential risk of misdiagnosis when a single modality is used. This finding prompted us to realize that a single ultrasound modality is sufficient to meet the diagnostic needs of complex cutaneous and soft tissue tumors. Looking forward, CEUS and SWE are gradually being integrated into clinical ultrasound workstations, and their application has become increasingly standardized. We are confident that expanding the sample size and collecting more comprehensive multimodal ultrasound data will enable us to further verify their synergistic effect in the diagnosis of JXG. We look forward to the multimodal ultrasound technology providing more accurate and reliable evidence for clinical diagnostic and treatment decision making.

The skin manifestations of JXG in infants and young children lack specificity and are easily confused with various skin and subcutaneous lesions under naked-eye observation, which significantly increases the risk of clinical misdiagnosis. Therefore, in clinical differential diagnosis, it is necessary to combine the ultrasonic imaging characteristics and conduct a comprehensive judgment on the basis of the clinical phenotype of the lesion to improve the diagnostic accuracy. JXG needs to be differentiated from infantile hemangioma (IH). IH is usually associated with a bright-red or violaceous cutaneous discoloration. Both JXG and IH lesions are located mainly in the skin, dermis, and/or superficial subcutaneous tissue. The characteristic ultrasound feature of IHs [24] is uniformly or slightly heterogeneous echogenicity and clear boundaries. In CDFI, the typical manifestation of IH is abundant branching or reticular blood flow signals, which is its core distinguishing feature from JXG. JXG also needs to be differentiated from Langerhans cell histiocytosis (LCH). The ultrasound manifestations of LCH are mostly hypoechoic masses with ill-defined boundaries and irregular shapes, which can involve the deep dermis and subcutaneous tissue. Some lesions can invade adjacent bone tissue, resulting in characteristic manifestations such as bone defects and hyperechoic bone changes. LCH lesions often have heterogeneous internal echogenicity and may present with cystic changes. CDFI shows moderately abundant blood flow signals within the lesions, mostly in the form of punctate or short rod-like signals [25]. Therefore, careful observation of calcification or cystic changes during ultrasound scanning and detailed evaluation of vascular morphology are crucial for accurate differential diagnosis.

## 5. Conclusions

In summary, the low incidence of JXG leads to frequent clinical misdiagnosis, particularly in patients with nonclassical clinical and imaging presentations. Ultrasonography serves as a reliable adjunctive modality for differentiating JXG. When yellowish-orange cutaneous lesions are identified in infants or young children, the detection of a homogeneous, clear boundary hypoechoic lesion confined to the dermis and superficial subcutaneous tissue should raise strong suspicion of JXG. Multimodal ultrasound imaging including CEUS and SWE may further improve the diagnostic confidence of JXG, especially for atypical cases.

## Figures and Tables

**Table 1:** The conventional ultrasound manifestations of all included patients.

Case	Gender	Age	Location, Solitary/Multiple, Shape	B-Mode					Color Doppler Flow Imaging (Internal Vascularization and/or Peripheral Vascularization)	Power Doppler Imaging (Internal Vascularization and/or Peripheral Vascularization)
				Size (mm)	Echogenicity	Border	Shape	Homogeneous/Heterogeneous		
1	male	4 months	Right shoulder, solitary, round	6 × 2 × 2	Hypoechoic	clear	regular	Heterogeneous	Internal vascularization	Internal vascularization, peripheral vascularization
2	female	2 months	Right shoulder, right back, multiple, round	38 × 15 × 34	Hypoechoic	clear	regular	Heterogeneous with septations	Internal vascularization	Internal vascularization, peripheral vascularization
3	male	8 months	Left neck, solitary, round	38 × 15 × 28	Hypoechoic	ill-defined	irregular	Homogeneous	Internal vascularization	Internal vascularization
4	male	11 months	Head, multiple, round	17 × 9 × 16	Hypoechoic	clear	regular	Homogeneous	Internal vascularization	Internal vascularization, peripheral vascularization
5	male	7 months	Right thigh, solitary, oval	8 × 5 × 9	Hypoechoic	clear	regular	Homogeneous	Internal vascularization	Internal vascularization
6	male	5 months	Lower part of the lower lip, solitary, round	6 × 4 × 6	Hypoechoic	clear	regular	Homogeneous	Neither	Internal vascularization
7	female	7 years	Left face, solitary, oval	24 × 6 × 23	Hypoechoic	clear	regular	Homogeneous	Internal vascularization	Internal vascularization, peripheral vascularization
8	female	3 years	Right face, solitary, oval	8 × 6 × 7	Hypoechoic	clear	regular	Homogeneous	Neither	Internal vascularization
9	male	7 months	Left ear, solitary, oval	10 × 6 × 8	Hypoechoic	clear	regular	Homogeneous	Neither	Neither
10	male	7 months	Left forehead, solitary, round	6 × 2.7 × 7	Hypoechoic	clear	regular	Homogeneous	Internal vascularization	Internal vascularization
11	female	3 years	Left back, solitary, round	15 × 6 × 16	Hypoechoic	clear	regular	Homogeneous	Internal vascularization	Internal vascularization, peripheral vascularization
12	female	3 years	Bilateral occipital region, multiple, oval	6.4 × 2.3 × 7.4	Hypoechoic	clear	regular	Heterogeneous	Neither	Neither
13	female	48 years	Left breast, solitary, oval	11 × 4 × 8	Hypoechoic	ill-defined	irregular	Heterogeneous with calcification	Neither	Neither
14	female	8 months	Right face, solitary, oval	15 × 7 × 9	Hypoechoic	clear	regular	Homogeneous	Neither	Internal vascularization

## Data Availability

The datasets presented in this study can be found in online repositories. The names of the repository/repositories and accession number(s) can be found in the article.
